# Delivery from the sky: investigating visual cues to communicate robot intentions in simulated public spaces

**DOI:** 10.1038/s41598-026-36451-z

**Published:** 2026-03-04

**Authors:** Shiva Nischal Lingam, Sebastiaan M. Petermeijer, Mohammad Obaid, Marieke Martens

**Affiliations:** 1 Royal Netherlands Aerospace Center, Amsterdam, The Netherlands; 2 Eindhoven University of Technology, Eindhoven, The Netherlands; 3 Chalmers University of Technology, Gothenburg, Sweden; 4 University of Gothenburg, Gothenburg, Sweden; 5 TNO, Helmond, The Netherlands

**Keywords:** Human-drone interaction, Public user, Delivery applications, Robot intentions, HMIs, Human behaviour, Aerospace engineering

## Abstract

As robots such as drones begin delivering packages from the sky in public spaces, humans will interact as recipients. Clear communication of a drone’s vertical motion and delivery intentions is essential to reduce feelings of uncertainty and build trust among the public. This study investigates how visual cues, including delivery methods and interfaces, communicate a drone’s drop-off and take-off intentions and affect recipients’ uncertainty in a simulated public environment. Through a video-based online questionnaire, 150 participants viewed scenarios where a drone delivered a package either by landing or via a cable-drop mechanism, each presented with or without visual interfaces such as onboard lights, a display, or ground projection. Participants rated the scenarios for uncertainty, understandability, predictability, trust, and convincingness, and provided qualitative feedback. Results show that visual interfaces improved participants’ ability to predict drone actions, increased certainty in approaching the drop-off spot, and improved trust. While lights posed challenges with visual clarity, both display and projection interfaces conveyed vertical motion and delivery intentions effectively. Projection was particularly recommended, as it marked the drop-off spot on the ground and a safety boundary. We discuss the implications of our findings beyond delivery scenarios, considering broader public space interactions with robots operating in vertical planes, and highlight the need for validation through real-world experiments.

**Supplementary Information:**

The online version contains supplementary material available at 10.1038/s41598-026-36451-z.

## Introduction

Robots, such as drones, are rapidly advancing and are expected to play an increasingly integral role in public life, particularly in package delivery. Drone delivery companies are using drones to transport medical supplies and food worldwide^1^. Such drones will interact with humans during the delivery of packages. Herdel et al.^2^ identified delivery as the most prominent application of Human-Drone Interaction (HDI; a sister domain to Human-Robot Interaction (HRI)) in their review of 217 studies spanning 33 domains, surpassing applications like photography and surveillance. Moreover, experts predict delivery will dominate public-space applications for HDI in the next decade^3^, highlighting its growing relevance and the frequency of interactions between drones and humans. As drones enter public spaces for deliveries, they engage with humans, for instance, as recipients who order packages. A significant challenge in public space HDI is to manage uncertainties experienced by humans^3^.

### Feelings of uncertainty and the need to communicate robot intent in HDI

Recipients may feel uncertain about a drone’s intentions, potentially due to unfamiliarity with drone technology, delivery processes and the lack of cues provided by the current drone technologies. Lingam et al.^4^[pg. 2] defined (feelings of) uncertainty in HDI as “a state of doubt experienced by users when interactions with drones deviate from the expected, leading to a loss of understanding of the drone’s intentions or its next actions,” which can affect decision-making^5^ and trust in automation^6^. In the literature, uncertainty is commonly measured using Likert scales^4,8^. The constructs of understandability, predictability, and trust are central to defining uncertainty in HDI^4^. Understandability refers to the degree to which the drone’s actions are easily comprehensible, whereas predictability reflects the extent to which the robot (e.g., drone) actions align with users’ expectations^10^. Higher levels of understandability and predictability are expected to be associated with lower uncertainty. For example, recipients who can understand and predict drone flight behavior typically experience low uncertainty^4^. Uncertainty is closely related to trust. Trust involves an individual’s willingness to rely on an agent (e.g., drone) to achieve a goal under situations of uncertainty^6^. Empirical evidence suggests that designing drone systems to minimize uncertainty can affect users’ trust^4^ and improve HDI across cultural contexts^11^. Designing drones to minimize human-felt uncertainty is thus a reasonable goal to improve HDI.

Clear communication of a drone’s intentions presents a possible approach to reducing uncertainty and improving usability, safety, trust and public acceptance^3,4,9,12–14^. While prior studies emphasize the importance of clearly communicating drone intentions, such as delivery timing, landing location, and approach direction, to support recipient feelings of easiness, certainty, and safety in approaching the package^4,15,16^, there is limited empirical evidence on how drones should convey these intentions to reduce uncertainty during delivery.

### Communication cues for HDI

Various studies^3,4,13,17–25^ explored cues, including movement and Human-Machine Interfaces (referred to as ‘interfaces’) and provided design recommendations for communicating drone intentions and improving HDI experience. Szafir et al.^23^ and Cauchard et al.^20^ demonstrated how flight behavior can be used to communicate a drone’s intentions and influence recipients’ perceptions. For instance, Szafir et al.^23^ demonstrated that drones using an arc trajectory were perceived as safer and more intuitive to interact with than drones executing a straight trajectory. Beyond flight behavior, delivery methods can also serve as cues to signal intent of a delivery drone^4^. Drone delivery companies use various delivery cues to implicitly communicate drop-off intentions^26–28^. A straight descent signals an imminent landing^17,18,21^, while hovering with a cable-suspended package indicates an aerial delivery^28^. Beyond signaling intent, delivery methods (e.g., drone landing, cable drop-off) could influence user perceptions of uncertainty, trust, and safety. Lingam et al.^4^ found that hovering drones delivering via cable were perceived as safer and more trustworthy than landing drones. Beyond implicit cues like delivery methods, users emphasized the need for interfaces to clearly communicate the drone’s intentions, particularly regarding the location and moment when the package will be released^4,9^.

Several studies have studied alternative cues to the movements of robots, such as interfaces incorporating lights, sound, displays, and projections, for explicitly communicating robot intentions^7,13,24,25,29–32^. Hetherington et al.^31^ found that projected arrows (i.e., left, right) from a ground robot enhanced motion legibility more than the lights alone, and that the absence of interfaces was found socially unacceptable. Szafir et al.^24^ showed that LEDs improved users’ ability to predict a drone’s flying direction to the left or right. Walker et al.^25^ demonstrated that displaying an arrow on an augmented head-mounted display to indicate the drone’s flight direction was perceived as clear and improved task efficiency for humans collaborating with the drone on a logistics management task. Herdel et al.^30^ found that using a display attached to a drone to communicate appropriate emotions positively influences recipient trust, compared to displaying inappropriate emotions. Wengefeld et al.^32^ used projections to indicate safety distances, guiding pedestrians and preventing them from getting too close. Cauchard et al.^19^ demonstrated that projection technology can be integrated into drones to communicate task information (e.g. photography) and prompt users to perform specific gestures to trigger actions (e.g. drone takes pictures). Obaid et al.^13^ found that light projections and audio cues were effective in promoting environmental cleanup, with participants responding positively. These studies recommend using interfaces to communicate robot intentions, primarily focusing on ground-level or lateral communication.

### Research gap

As drones enter public spaces for deliveries and operate in vertical spaces, their intentions change across interaction stages such as drop-off and take-off^9^. For instance, drones descend during drop-off and retract during take-off. These shifting intentions can create uncertainty for the recipient in interpreting the drone’s intentions across the stages and when to approach the drop-off spot. These uncertainties were not addressed by previous HRI studies. A clear research gap exists in how to represent the internal state of a delivery drone and effectively communicate the stage transitions, thus reducing uncertainty and enabling the acceptance of drone technologies in inhabited environments. This has motivated us to address the gap by investigating how visual cue representations can communicate a delivery drone’s internal states, which then can lead to improving trust and safety in HDI. The following outlines the details of our approach and study to address this research gap.

### Study focus

One approach to handling uncertainty felt by the user in HDI is to signal a drone’s intentions—particularly in vertical motion—through visual cues, allowing users to anticipate its actions and respond accordingly. This was investigated in our study using visual cues, including delivery methods and visual interfaces. This research examines two delivery methods: (1) a drone landing to deliver a package, and (2) a drone hovering above eye level and dropping the package using a cable. The visual interfaces include: (1) a screen display mounted on the drone, (2) ambient lights attached to the drone, and (3) a ground projection. Specifically, this research aims to answer the question: *How do visual cues*,* in terms of delivery methods and visual interfaces*,* affect recipients’ uncertainty during drone delivery in public spaces?* The study focuses on the drop-off and take-off stages, with a preparation stage before each to ensure stabilization and safety^4^.

We chose to focus on visual cues, as they have been frequently mentioned by the users as the relevant feedback method for delivery drones to communicate in public spaces, based on a user-centered interview and design study^9^. Moreover, visual cues are the primary mode of communication in public environments such as roads; for example, traffic lights and vehicle indicator lights are commonly used to convey information. Audio interfaces were excluded from the study due to potential clarity issues caused by the propeller noise of large delivery drones and the negative perception of drone sounds^33,34^. Drone sounds in public spaces can mask environmental sounds, increase perceived loudness^35^ and disturb bystanders, who are passersby and not recipients. For instance, Callanan et al.^36^ observed that drone sounds interfere with verbal communication among people in the vicinity.

To assess user uncertainty regarding these visual cues, an online questionnaire with video-based scenarios was conducted. Uncertainty was evaluated using constructs such as understandability, predictability, and trust. In addition, we measured the convincingness of the visual cues, to understand how certain recipients felt about approaching the drop-off spot. Convincingness influences user decision-making and has been used as an indirect measure of trust in HRI^37,38^. Implications can be drawn on whether designing visual cues for reduced uncertainty convinces users to feel certain in their approach intentions to collect the package. The main contributions of our study are:

1. We investigate ways to reduce user perceived uncertainty and improve their trust in delivery drones by studying visual cue representations in public spaces during aerial deliveries.
2. We provide a list of recommendations for the design of visual cues on robots operating in vertical space.

## Methods

Video-based online surveys have been commonly used in HRI literature^39,40^ to examine user perceptions, including interactions with Automated Vehicles (AVs) and drones that are in virtual form^30,41,42^. Video-based surveys provide a methodological approach that is scalable, reproducible, and safe for both HRI^42^ and HDI research^30,41,43^. Prior evidence indicates moderate to high agreement between participants’ ratings in live and video-based HRI studies, with respect to participant preferences and perceptions^44^. As a part of PhD research, Tabone^45^ conducted a video-based survey^42^, a laboratory study^46^, and an outdoor experiment^47^ to evaluate visual cues for AVs. His findings showed that subjectives measures, such as understandability and convincingness, were highly correlated between video-based and laboratory studies, and the results across the three methods were comparable. Similarly, Honig et al.^48^ found that laboratory studies and video-based surveys yielded comparable outcomes. The authors suggested the use of video-based surveys for HRI design and evaluation. Furthermore, it is challenging to conduct real-world experiments with delivery drones in public spaces in the Netherlands (location of study). The current safety regulations^49^ require drones weighing more than 500 g to maintain a minimum distance of 50 m. These regulatory constraints and prior research informed our decision to adopt a video-based online study.

In this study, participants completed an online survey on Qualtrics (https://www.qualtrics.com/), where they watched and rated drone-related videos based on several criteria. The videos showed a drone delivering a package to the participant, using visual cues such as visual interfaces (i.e., display, lights, and projection) and delivery methods (i.e., drone landing, and cable drop). The visual interfaces required to develop the videos were designed through an iterative user-centered approach.

### Design of visual interfaces

The visual interfaces were developed through a series of focus groups with seven participants (3 male, 4 female; aged 25–32, M = 28.5, SD = 2.23) and a professional designer. Participants were from three cultural backgrounds: Indian (4), Chinese (2), and Dutch (1). The participants had limited experience with drones and no prior experience with delivery drones. Current safety regulations in the Netherlands^49^ restrict the use of delivery drones in proximity to individuals in public spaces. Delivery drone technology remains in the testing phase, primarily limited to hospital-to-hospital operations in the Netherlands^50^ rather than deployment in public environments. Consequently, the proportion of individuals with prior experience interacting with delivery drones is expected to be very low.

**Fig. 1:**
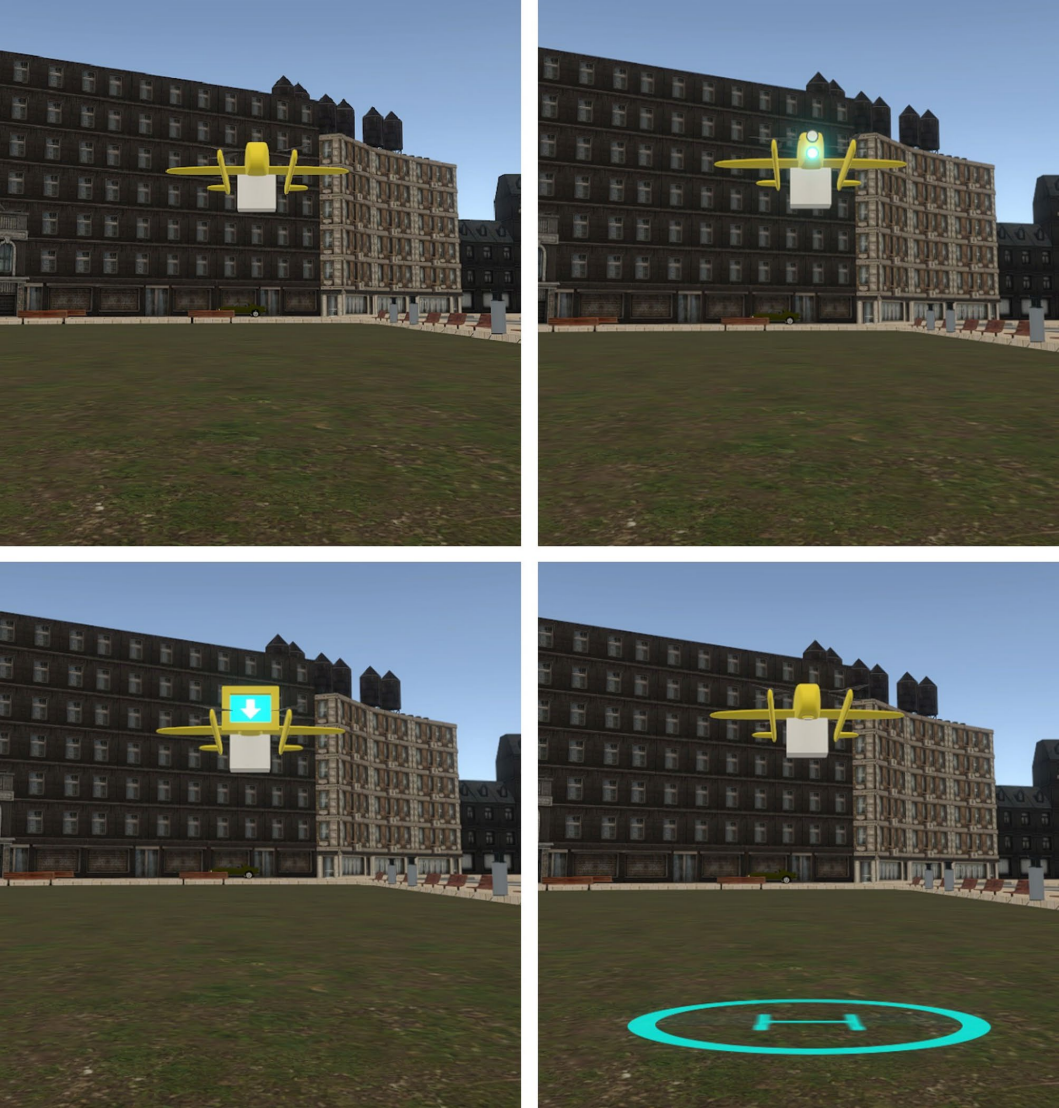
Illustration of 4 interface variants, namely Baseline (top-left), Lights (top-right), Display (bottom-left), and Projection (bottom-right). The virtual 3D models are created using Blender (version 4.4; https://www.blender.org/) and Unity 3D (version 2021.3.22f1; https://unity.com/).

Participants were presented with a scenario in which a drone enters public space from the sky to deliver a nearby package. They were asked to propose ideas for three interfaces—Display, Lights, and Projection—focusing on the preparation, drop-off, and take-off stages. We avoided color, text or culture-specific symbols and instead encouraged the participants to use intuitive metaphors and light animations, minimizing the need for users to learn new “languages” or associate their “own” meanings. The professional designer translated their ideas into virtual models using Blender (version 4.4; https://www.blender.org/) and Unity 3D (version 2021.3.22f1; https://unity.com/*)* in a Virtual Reality (VR) environment. Concepts were iteratively refined based on participant feedback until a general consensus was reached, with the moderator (first author) facilitating discussions to resolve any disagreements and ensure the final designs met the participants’ needs. Cyan was chosen for the light animations as it was considered a neutral color in the literature, without inherent associations with robotic states, such as those used for automated vehicles on public streets^29,51,52^. The developed interface concepts are as follows:

1. Display [see Fig. 1 (bottom-left)]: A display on the drone’s “face” used animated arrows to show the drone’s direction along the vertical plane, inspired by elevator displays and previous research on directional arrows for intent communication of the robots^48,52^. The arrows were animated in the direction of movement during the drop-off and take-off stages and remained static during the preparation stage.
2. Lights [see Fig. 1 (top-left)]: A pair of vertically arranged LED lights on the drone’s “face” indicated the direction of the package during drop-off or take-off. The bottom light blinked during the drop-off stage, the top light blinked during the take-off stage, and both blinked during the preparation stages. The design for direction indication (going down or up) with blinking lights was inspired by existing mental models of motor vehicle turn signals^31,53^.
3. Projection [see Fig. 1 (bottom-right)]: A ground projection was used to signal the package drop-off on the ground. Participants suggested using the “H” symbol enclosed within a large circle to resemble existing mental models of a helicopter landing pad. The projection marked a spot on the ground with an “H” to indicate the drop-off stage and the surrounding safety zone with a constant circular ring. The “H” projection blinked during the drop-off, and the circular ring blinked during the take-off stage. The projections remained static during the preparation stages.

The Baseline condition [no visual interface; see Fig. 1 (top-left)] was included for comparison purposes to assess the effect of visual interfaces’ presence on user responses.

### Scenarios

**Fig. 2:**
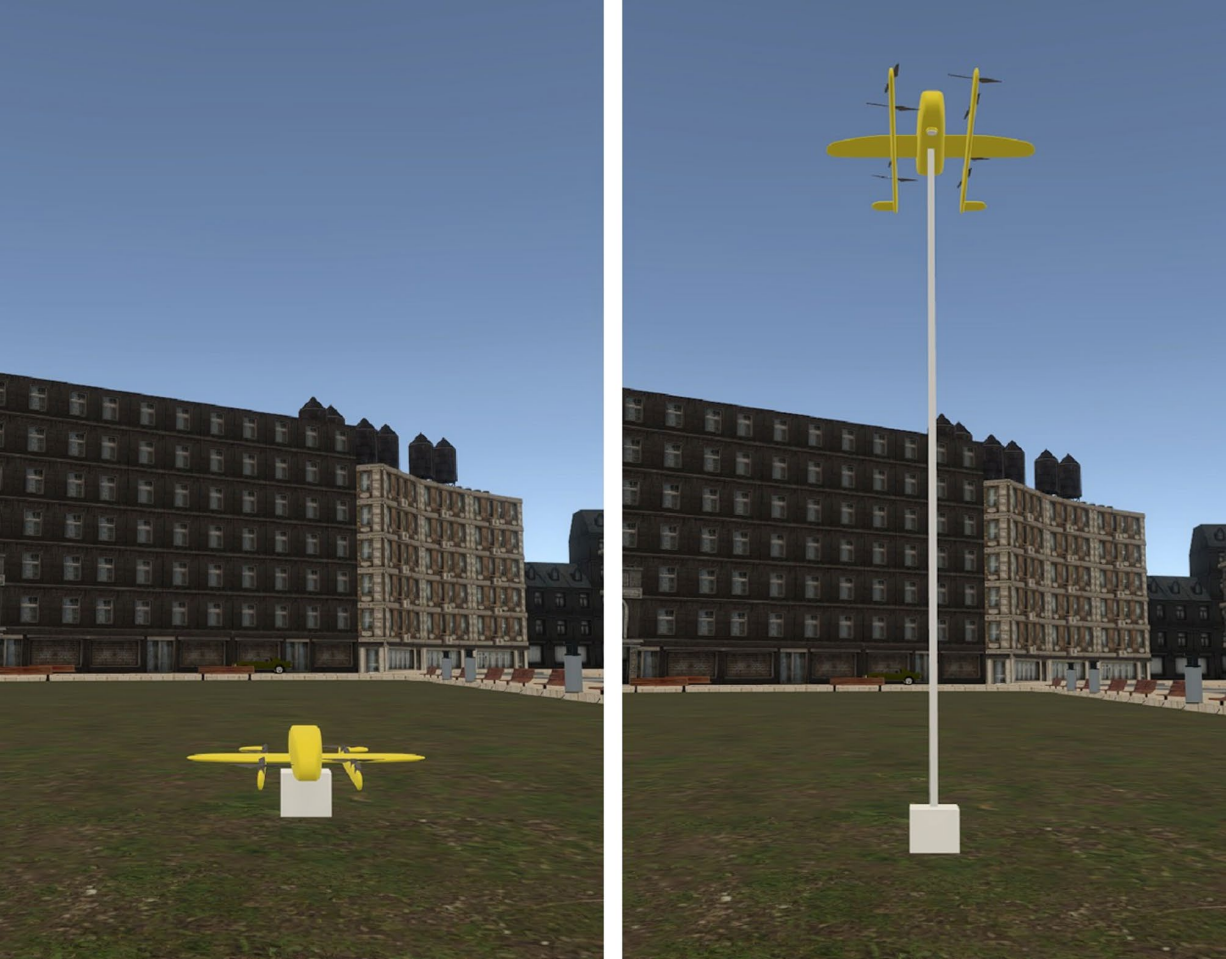
The two delivery methods, namely Land (left) and Cable (right). The virtual 3D models are created using Blender (version 4.4; https://www.blender.org/*)* and Unity 3D (version 2021.3.22f1; https://unity.com/).

**Fig. 3:**
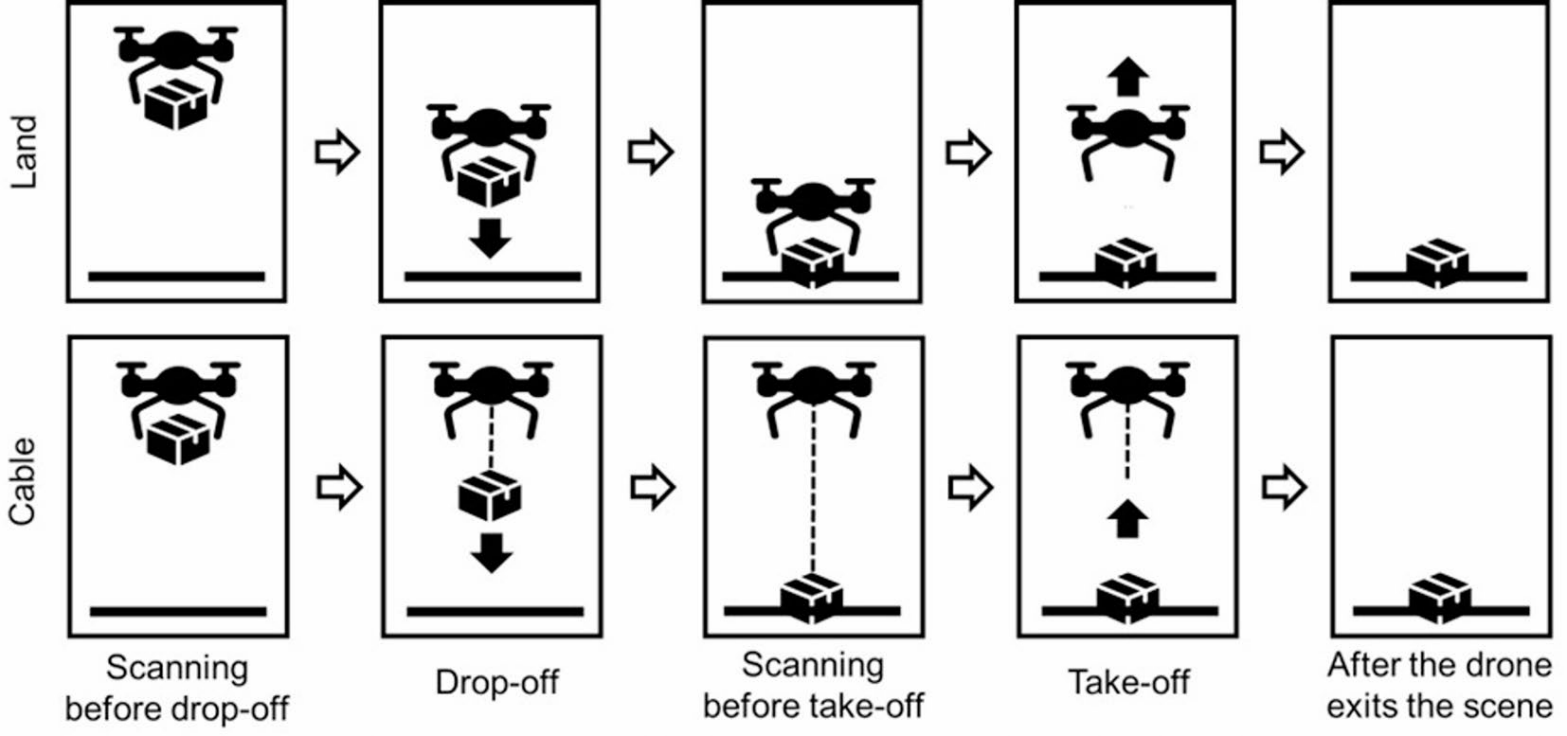
Stages of interaction experienced by the participant for the two delivery methods, namely Land (top) and Cable (bottom).

The 4 × 2 within-participant design study included two independent variables. The within-participant variables were interface, with four variants (Baseline, Display, Lights, and Projection; see Fig. 1), and delivery method, with two variants (Cable, Land; see Fig. 2). The drone entered the scene by descending to 7 m and hovering^4^. The hovering signaled the intent to prepare for delivery and scan the surroundings for safety during the preparation stage. Next, the drone descended itself (in Land method) or lowered the rope (in Cable method) to place the package on the ground in the drop-off stage. Next, the drone signaled its intent to prepare for departure by either initiating its propellers while on the ground (in Land method) or hovering (in Cable method), scanning the surroundings before take-off. In the take-off stage, depending on the condition, the drone either ascended (in Land method) or the rope retracted (in Cable method). The hover and scan intentions represented the preparation stage, while the drop-off and take-off intentions represented the action stages. The preparation stage happened before each action stage. The stages of interaction are illustrated in Fig. 3 for the two delivery methods.

### Videos and experiment setup

A total of 8 videos (at 30 frames per second; see Supplementary material > > Scenarios.mp4 to view videos) were created to depict a drone performing delivery functions, incorporating different interfaces and delivery methods. Of the 8 videos, 4 showed the drone delivering using a cable rope, while the remaining 4 depicted delivery by drone landing on the ground.

The public space environment was adapted from a previous VR study on delivery drones^4^. The drone entered the scene by descending to a height of 7 m and started the drop-off with either of the two delivery methods. Each drop-off and take-off stage lasted for 10 s, and the preparation stage for 5 s. After retracting, the drone ascended and exited the scene. Each video had a total duration of 35 s. The videos were recorded from a fixed viewpoint of a recipient, positioned at a distance of 7 m from the drone^4^.

The design of the drone model (dimensions: 1.3 m × 1.0 m × 0.4 m), its movements, and propeller noise were adapted from Lingam et al.^4^. The drone’s movements were replicated using proportional–integral–derivative (PID) controllers to approximate real-world flight behavior while maintaining experimental control. Propeller noise was synchronized with the PID controllers and the drone’s movements in an attempt to replicate the audiovisual feedback of a drone operating in a real environment.

The quantitative parameters of the visual interfaces were calibrated based on a pilot study. The relative luminance intensity of the visual interfaces was set to 12 in contrast to the background lighting in the virtual environment, the LED and the projection blinking rates were set to 2 blinks/s, and the arrow animation speed was set to 0.65 cycles/s.

### Measures

The measures included both Likert scale questions and qualitative responses. After each video, participants evaluated visual cues (i.e., interfaces and delivery methods) using questions (see Table 1) adapted from literature on uncertainty^4^, understandability, predictability, trust^54^, and convincingness^42,55^. Responses were recorded on a seven-point Likert scale (1: strongly disagree; 7: strongly agree).

**Table 1:** Questions on uncertainty, understandability, predictability, trust and convincingness towards the cues, representing the delivery methods and the interfaces.

Scale	Item
Uncertainty	The cues made me feel uncertain about the drone’s action.
Understandability	The cues, intended to show the drone’s actions, were clear to me.
	I was able to understand the drone’s actions based on the cues.
Predictability	The cues I received acted unpredictably.*
	It was difficult to identify what the cues would show next about the drone’s actions.*
Trust	I trusted the cues to inform me of the drone’s actions.
	I relied on the cues to inform me of the drone’s actions.
Convincingness	The cues made me feel certain to approach the drop-off spot.

* inverse statement.

Following the Likert-scale questions, participants were shown a picture of each stage and were asked to indicate the stage at which they would feel certain to approach the drop-off spot (referred to as participant approach intention), choosing from ‘scanning before drop-off’, ‘drop-off’, ‘scanning before take-off’, ‘take-off’, ‘after the drone exits the scene’, or ‘none of the above’ options. The participant approach intention measure provided additional insight into the convincingness measure by reflecting on when the recipients intended to approach the drop-off spot. Participants were provided with a free text area to elaborate on their subjective ratings and preferences for each cue.

At the end of the questionnaire, participants saw pictures of the 8 scenarios and ranked them by preference, from 1 (least preferred) to 8 (most preferred). The participants were then asked to provide textual explanations for their highest and lowest-ranked choices.

### Procedure

The study was conducted in English using an online questionnaire. Informed consent was obtained from all participants prior to their participation. The survey began by outlining the study’s purpose, focusing on addressing feelings of uncertainty through the use of visual cues. Participants were recruited using the Prolific platform (https://www.prolific.com/*).* In line with the past HDI studies^30,56^, the selection criteria for our participants included a minimum of 100 prior successful submissions, an approval rate of previous submissions ≥ 97%, native English proficiency, and residency in the United States. Participants could access the survey only on a laptop or desktop device and with audio enabled. The concept of delivery drones in a public park was briefly introduced, adapted from Lingam et al.^9^. The recipient’s role was explained, along with how drones communicate their delivery intentions to the public through interfaces and delivery methods. Participants were informed about the stages and that communication occurred via static or animated lights and signals. However, specific design elements (e.g., the projected circle) were not explained to allow for intuitive interpretation and minimize bias.

Participants provided demographic information, including gender, age, country of residence, education level, and attitudes toward technology interaction^57^. They also reported their prior experiences with drones. Each scenario was presented across two survey pages: the first for the video and the second for the corresponding assessment questions. Before viewing each video, participants were instructed to maximize the video to full screen and set the volume to maximum. After watching the video, participants proceeded to the next page where the assessment questions were presented. The presentation of the scenarios was randomized to minimize order effects. At the end of the questionnaire, participants rated their preference for each concept and provided rationale for their highest and lowest preferences. Reliability check questions were included throughout the survey to verify participants’ understanding of the context and attentiveness^51^. Several questions verified whether participants (1) responded from the perspective of the recipient and (2) understood that the cues represented the drone’s delivery intentions, as well as whether they were paying attention while completing the survey. Participants were required to pass all the check questions for their responses to be included in the analysis. The entire experiment took approximately 30 min, and participants were compensated with £4.25. The study procedure was approved by the Ethical Review Board of Eindhoven University of Technology and was conducted in accordance with relevant ethical guidelines and regulations.

### Participants

After excluding 10 participants due to technical issues or multiple failed attention checks, the sample consisted of 150 participants (77 female, 73 male), aged between 18 and 64 years (*M* = 39.5, *SD* = 10.4). With regards to the highest level of education, 4 participants had completed doctoral education, 31 Master’s or equivalent education, 55 Bachelor’s or equivalent education, 48 Secondary education, and 12 Primary education.

Most participants (*n* = 148) had previously seen a drone either in media or real life, and 27 reported owning a drone. In addition, 141 participants had seen a drone from a distance, 121 had observed a drone flying in close proximity, and 44 had experienced piloting a drone, whereas 9 had never encountered a drone in reality. Overall, participants reported a generally positive attitude toward technology interaction (*M* = 4.1, *SD* = 1.3).

### Analyses

The Likert-scale data (*N* = 150) violated the normality assumption for all the 6 measures: uncertainty (*W* = 0.86, *p* < 0.001), understandability (*W* = 0.88, *p* < 0.001), predictability (*W* = 0.90, *p* < 0.001), trust (*W* = 0.90, *p* < 0.001), convincingness (*W* = 0.88, *p* < 0.001), and preference (*W* = 0.93, *p* < 0.001). The data were analyzed using non-parametric methods. The Likert scale data was evaluated with Aligned Ranks Transformation ANOVA (ART-ANOVA)^58^, which has been commonly applied in previous HCI and HDI studies^59–61^. This method involves applying the aligned ranks transformation procedure to the Likert-scale data, followed by testing with repeated-measures factorial ART-ANOVA^58^. Post-hoc pairwise comparisons were conducted for significant main and interaction effects using the extended ART-Contrasts procedure^62^, with Bonferroni correction (hereafter referred to as post-hoc tests) applied to account for multiple comparisons. Only significant post-hoc results are reported for brevity. Spearman correlation was reported between uncertainty and other Likert scale measures, such as understandability, predictability, trust, and convincingness, to examine the relationship between uncertainty and the above constructs, and their role in interpreting uncertainty. All statistical tests conducted were two-tailed with an alpha level of 0.05. Due to a large sample size, even small within-subject differences between the visual cues were strongly significant.

The data on participant approach intention, excluding “none of the above” responses, were analyzed using a Chi-square test. The dataset included 149 and 147 observations for the Cable and Land methods in the Baseline condition, and 149 and 148 observations for the Cable and Land methods in the Lights condition, respectively.

A total of 1,500 textual responses were collected across eight scenarios, as well as from the end-of-questionnaire questions regarding participants’ most and least preferred cues. A thematic analysis^63^ was conducted on the textual responses to examine participants’ subjective expressions. Two coders were employed for the analysis. The coders familiarized themselves with the data, then used MaxQDA software (https://www.maxqda.com/*)* to code and perform the analysis. The coding process was inductive and the codes were emergent from the data. Initial codes were developed based on raw quotations, and these codes were assigned to potential themes based on similarities, differences, and repetitions. The codes and themes were developed iteratively and were continually reviewed and refined against the original dataset. The interrater reliability, computed using Cohen’s *kappa*, was 0.87, indicating an excellent level of agreement.

## Results

### Likert scale measures

**Fig. 4:**
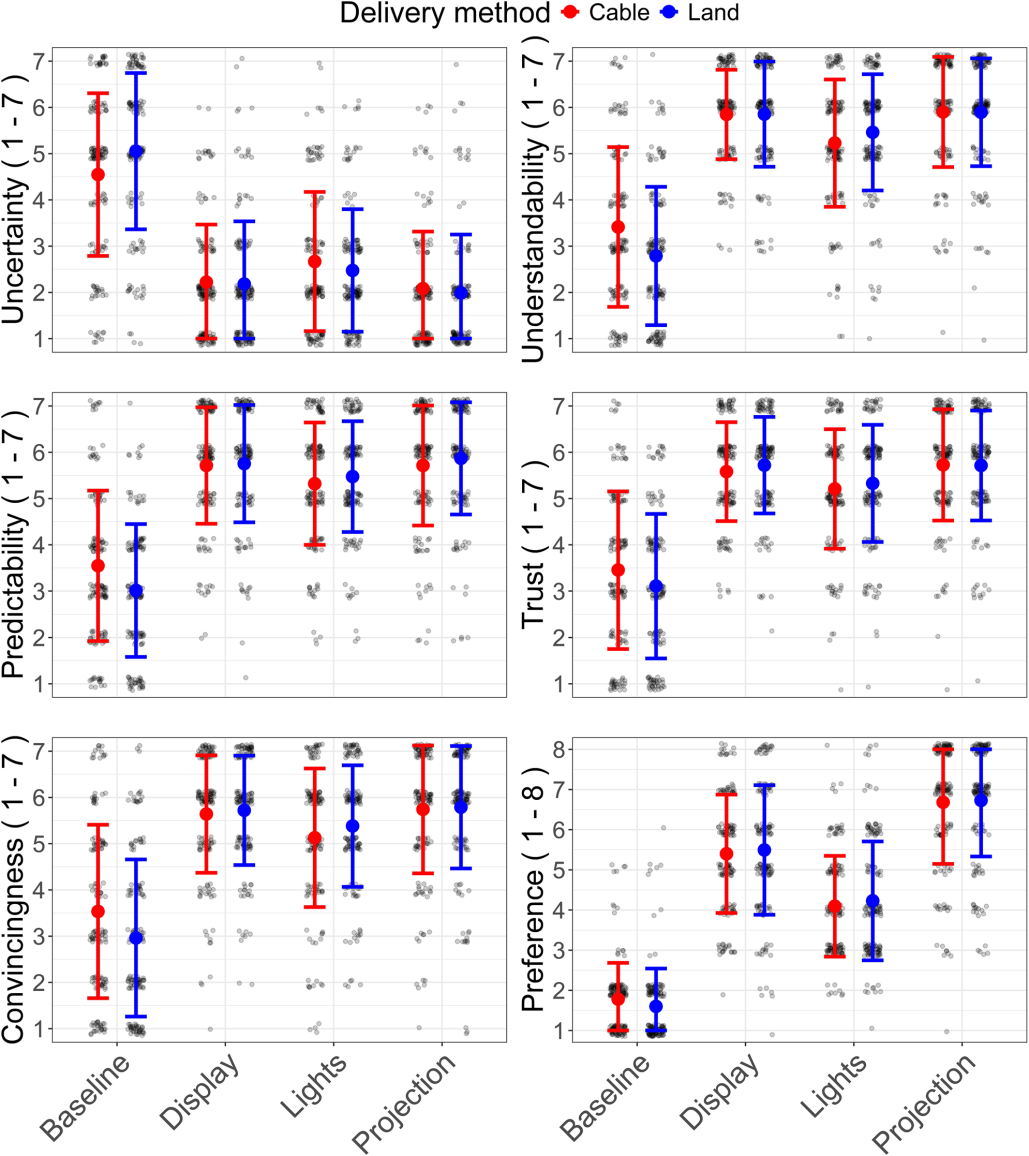
Mean and standard deviation of the uncertainty, understandability, predictability, trust, convincingness and preference measures for the 8 visual cues. The individual data points are overlaid. The Likert scale measures ranged from 1 (strongly disagree) to 7 (strongly agree), while preference was assessed on a rank-based scale from 1 (lowest preference) to 8 (highest preference).

Figure 4 presents the means and Standard Deviation (SD) of the 6 Likert scales across interface conditions and delivery methods. The Baseline condition, especially for the Land method, received the highest uncertainty scores and the lowest ratings for other measures.

ART-ANOVA was conducted to assess the effects of interface and delivery methods on the six Likert scales. The results (see Table 2) indicated significant main effects of the interface on all six Likert scales, with large effect sizes.

**Table 2:** ART-ANOVA results for the six likert scale measures, namely, uncertainty, understandability, predictability, trust, convincingness, and preference. *p* < 0.001 ***, *p* < 0.01 **, *p* < 0.05 *.

Measure	Effects	df	F	*p*	η_*p*_^2^
Uncertainty	Interface	3	258.43	< 0.001***	0.426
	Delivery method	1	2.92	0.088	0.003
	Interface* Delivery method	3	4.5	0.004**	0.013
Understandability	Interface	3	337.59	< 0.001***	0.493
	Delivery method	1	8.36	0.004**	0.008
	Interface* Delivery method	3	9.68	< 0.001***	0.027
Predictability	Interface	3	286.59	< 0.001***	0.452
	Delivery method	1	3.97	0.05	0.004
	Interface* Delivery method	3	4.9	0.002**	0.014
Trust	Interface	3	266.2	< 0.001***	0.434
	Delivery method	1	2.77	0.096	0.003
	Interface* Delivery method	3	2.78	0.04*	0.008
Convincingness	Interface	3	224.14	< 0.001***	0.392
	Delivery method	1	6.26	0.012*	0.006
	Interface* Delivery method	3	4.94	0.002**	0.014
Preference	Interface	3	662.41	< 0.001***	0.656
	Delivery method	1	< 0.01	0.93	< 0.001
	Interface* Delivery method	3	2.83	0.04*	0.008

Significant main effects of the delivery method were found for the Likert scales measuring understandability and convincingness, but the effect sizes were very small (see Table 2). There were significant interaction effects between interface and delivery methods for the six measures, but the effect sizes were small (see Table 2).

**Fig. 5:**
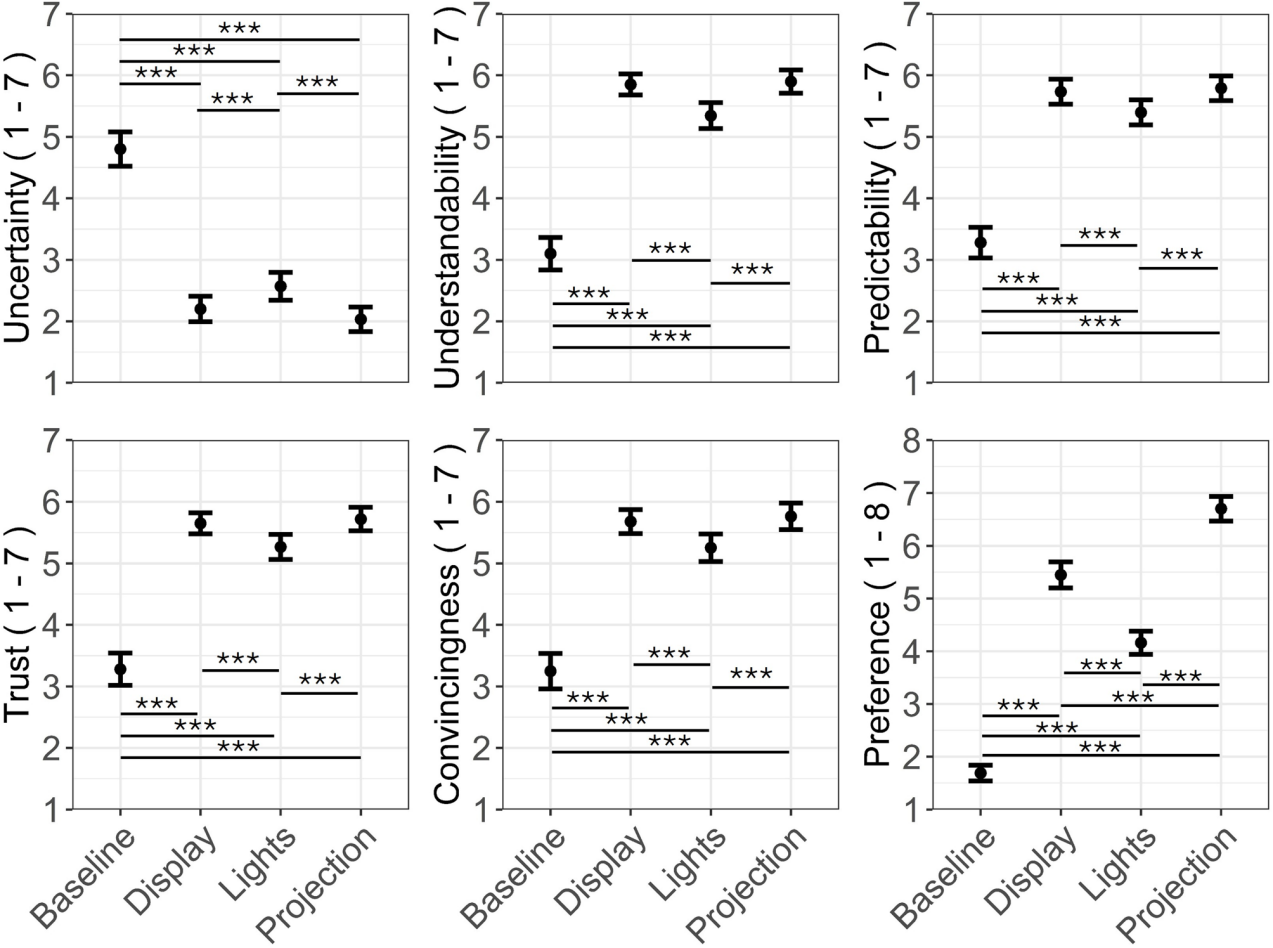
Mean and error bars of the four interfaces—Baseline, Display, Lights, and Projection—across the six Likert scale measures: uncertainty, understandability, predictability, trust, convincingness, and preference, combined for the two delivery methods. The Likert scale measures ranged from 1 (strongly disagree) to 7 (strongly agree), while preference was assessed on a rank-based scale from 1 (lowest preference) to 8 (highest preference). The error bars represent standard errors. Pairwise comparisons with significance, *p* < 0.001***, *p* < 0.01**, *p* < 0.05*.

Uncertainty scores in Fig. 5 were highest for the Baseline, followed by Lights, Display, and Projection. In contrast, understandability, predictability, trust, and convincingness scores were highest for Projection, followed by Display, Lights, and Baseline. Post-hoc pairwise comparisons (see Fig. 5) revealed significant differences in the mentioned six measures between Baseline and the three other interfaces, as well as between Lights and the remaining two interface conditions. Participants showed the highest preference for Projection, followed by Display, Lights, and Baseline. Post-hoc analyses for the preference measure indicated significant differences between all the interface pairs.

Post-hoc tests for the delivery method showed that Cable (*M* = 5.1; *SE* = 0.07) received somewhat higher understandability scores than Land (*M* = 5.0; *SE* = 0.07), *p* = 0.004. On similar lines, Cable (*M* = 5.01; *SE* = 0.07) received somewhat higher convincingness scores than Land (*M* = 4.96; *SE* = 0.07), *p* = 0.013.

Post-hoc tests for the interaction pairs (interface*delivery method) revealed that Baseline-Cable received significantly higher scores for understandability (*p* = 0.007), predictability (*p* = 0.031), and convincingness (*p* = 0.034) compared to Baseline-Land. Mean and SD of the Likert scales for the interaction pairs is shown in Fig. 4.

Uncertainty was strongly negatively correlated with understandability (*r* = -0.84, *p* < 0.001), predictability (*r* = -0.84, *p* < 0.001), trust (*r* = -0.75, *p* < 0.001), and convincingness (*r* = -0.76, *p* < 0.001).

### Participant approach intention

**Fig. 6:**
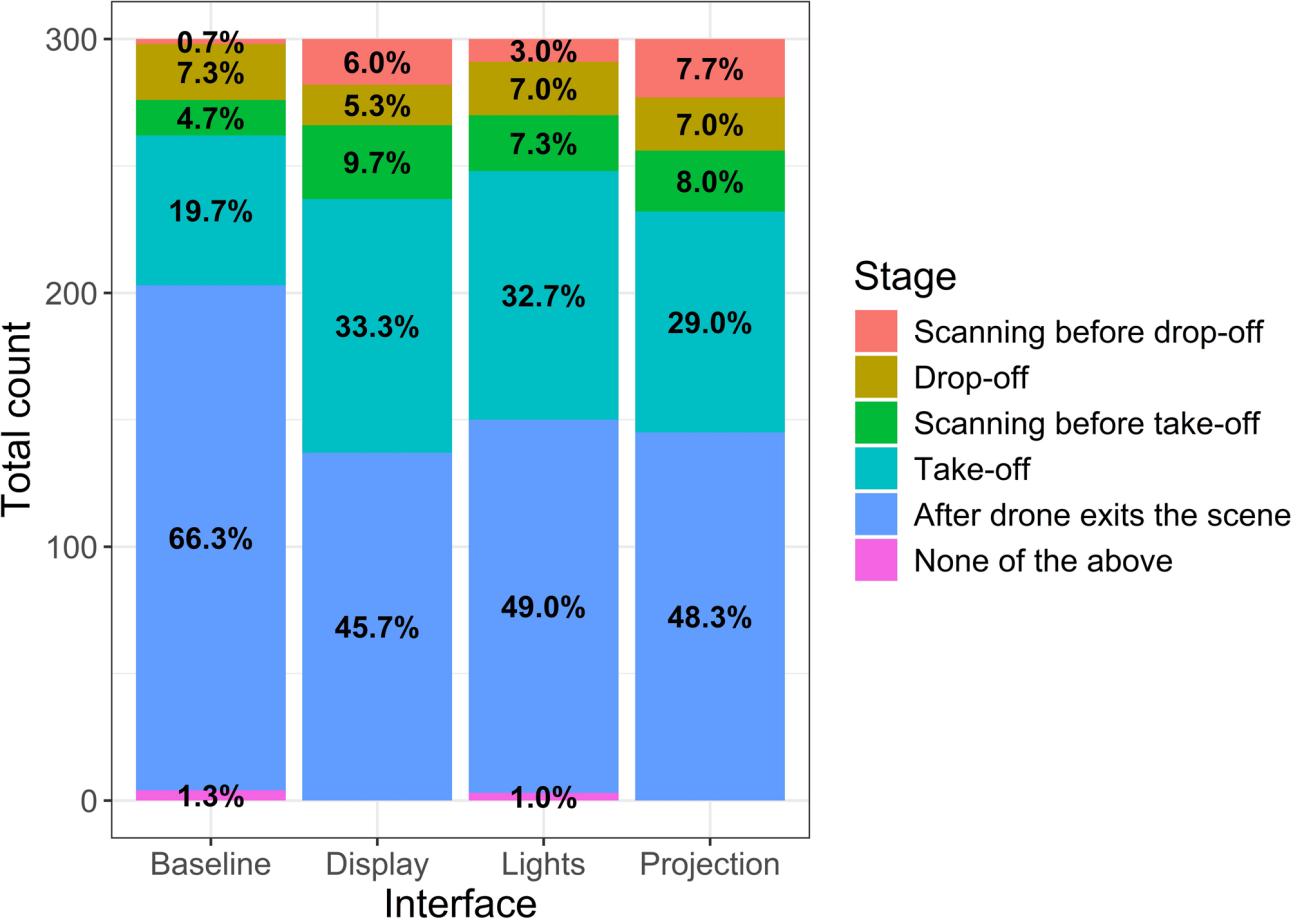
Proportion of participants indicating the stage at which they would approach the drop-off spot across the four interfaces (Baseline, Display, Lights, and Projection) and combined for the two delivery methods.

The proportion of participants reporting their approach intention did not significantly differ between the stages by the delivery method, X²(4, *N* = 150) = 2.1, *p* = 0.72. However, significant differences were observed between the stages across interfaces, X²(12, *N* = 150) = 54.39, *p* < 0.001. This indicates that the interface significantly affected participants’ choices when to approach the drop-off spot (see Fig. 6). In the Baseline condition, most participants indicated their intent to approach the package only after the drone had exited the scene, compared to when interfaces were present. Specifically, most participants indicated an intent to approach before drop-off and take-off, with Projection and Display leading, followed by Lights and Baseline.

### Textual responses

This section presents themes and quotes from the thematic analysis. The findings are presented alongside relevant participant quotes, denoted by ‘P’ followed by their ID number. The themes and codes are presented in Table 3.

**Table 3:** Themes corresponding to the codes extracted from the open-ended responses.

Need for explicit information on drone intentions	Reflection on interfaces	Reflection on delivery methods	Additional solutions to reduce uncertainty
Inadequate information makes it difficult to interpret drone intentions	Ease of interpretation and uniqueness of each interface	Preference between Cable and Land methods	Combination of visual interfaces
Effect of information inadequacy on recipient perception	Criticism for not providing information clearly	Safety concerns with the application of delivery methods in public spaces	Color coding visual interfaces
	Visibility concerns with the interfaces in public spaces		

#### Need for explicit information on drone in tentions

Most participants reported that the absence of explicit cues made it difficult to spot the drone’s entry into the public space and understand its delivery intentions. This lack of clarity raised confusion and uncertainty, demanded greater attention towards the drone, and raised discomfort and safety concerns:

“Since there were no (interface) cues, I couldn’t be certain what the drone was going to do or where it was going to land precisely.” (P67).

“No indication of any actions and it is dropping the package (with the cable) from the sky. This will definitely injure people in the area.” (P94).

#### Reflection on interfaces

Interfaces providing intention cues improved a majority of participants’ ability to understand drone actions, reduced uncertainty, increased perceived safety, and helped determine the appropriate time to approach. Participants provided both positive and negative feedback on each interface, offering insights into their applicability in public spaces.

Having the Display and Light interfaces on the drone itself “makes them easy to associate with the drone’s actions” (P124), especially for the Land method. Participants found the arrows on the Display to be clear, and natural for interpreting each stage of the process and the drone’s motion. Lights were considered simple and effective, though they required some visual scanning to interpret: “The static lights for scanning, and blinking lights for drop-off make sense. It may take a time or two to get used to what the lights mean, but overall it’s effective” (P40). The Projection was widely recognized for conveying the most comprehensive information: “direction (of motion), (safety) zone size (with circular ring) and placement (of package), hover or movement mode” (P106). Participants interpreted the blinking circle as an indication that the drone or the cable was retracting and that the disappearance of the circular projection implied it was safe to approach. The large circle with the “H” symbol was interpreted as indicating a drone landing on the ground, as participants noted that the symbols “were similar to a helicopter pad and everyone knows what that means” (P78).

The three interfaces were criticized for not providing explicit information on “(…) when it is ok to approach the package” (P103). Display and Lights were criticized for limiting visibility from different ground angles and requiring users to look up, particularly when the drone hovered above eye level during the Cable method. This raised concerns about safety and awareness in public spaces: “This (Display) seems dangerous if someone is walking by and not looking up” (P39). A few participants suggested replacing the “H” symbol in the Projection with text such as “delivering package” (P38) or “X” (P103), as they felt that “H” could be interpreted as indicating a landing drone rather than a package drop-off, even when using the cable drop method.

A few participants highlighted potential issues with “display size and glare” (P124), which could affect readability in public spaces with brighter light conditions. For the Lights, the main challenge was distinguishing between the top and bottom LEDs, particularly when the drone hovered above eye level. However, this issue was less pronounced when the drone descended: “At first, you’re unsure, then it becomes apparent what the signals are” (P48). Visibility concerns were raised for the Projection, particularly in varying lighting conditions or when obstructed by people or pets: “(Projection) could easily be blurred (by light conditions) or blocked from visibility” (P107).

#### Reflection on delivery methods

Participants were divided in their preference for delivery methods. Some participants considered the Cable method safer and offered more certainty than the Land method, as “you wouldn’t have people getting hit with propellers” (P39). Others expressed concerns about its reliability, including potential failures in “bad weather” (P55) or practical issues such as “cable mechanism malfunctions or a child or animal yanking on the cable” (P48). A few viewed the Land method as safer, highlighting its visibility and its ability to trigger natural avoidance reactions: “What if the package is coming down from the sky with the cable and a child stands under it? I feel like they are more likely to move if they see a big drone dropping down (landing)” (P46). The Cable method was preferred for “not invading the landing space” (P59) and delivering efficiently, while the Land method was favored for its simplicity and ease of execution.

#### Additional solutions to reduce uncertainty

Participants suggested combining interfaces (e.g., Display + Projection) as it “might be even more effective for ensuring clear communication in various viewing conditions” (P124). Additionally, participants mentioned the importance of color coding interfaces to differentiate between stages and indicate when it is unsafe to approach. For example: “A red circle would make it more obvious that you are not supposed to approach the drone or landing zone yet” (P95).

## Discussion

This online study examined how visual cues, including two delivery methods (drone landing and cable drop) and three visual interfaces (display, lights, and projection), affect recipients’ uncertainty about a delivery drone’s intentions in a simulated public space. Perceived uncertainty was assessed using Likert scales, including understandability, predictability, trust, and convincingness, alongside qualitative responses. Results showed a strong negative correlation between uncertainty and the mentioned Likert scale measures, indicating that the effects of uncertainty can be interpreted through these measures. Mixed-method analysis revealed that interfaces reduced uncertainty, with projection being the most preferred. However, no significant differences were found between the two delivery methods with the use of visual interfaces. Qualitative feedback highlighted the importance of explicit communication, particularly using displays to indicate package motion and projections to define drop-off spots and (un)safe zones. Visibility and safety concerns were raised with the practical implementation of the visual cues in public spaces.

Participants felt that delivery methods alone were insufficient cues and highlighted the need for interfaces to signal the drone’s actions to enhance recipient certainty and trust. Among the three interfaces, projection and display were associated with the lowest uncertainty and highest trust scores, with projection being the most preferred. Qualitative findings indicated that participants valued projection for conveying comprehensive information about the drone’s motion, drop-off location, and the surrounding circular safety zone, which were less apparent in the other interfaces. This finding broadens the potential use of projection interfaces in public space HRI, such as for marking garbage locations^13^, indicating task information^19^, displaying directional symbols^31^, and suggesting personal space for pedestrians to occupy near robots^53^. The display of arrows, inspired by elevator systems, reduced uncertainty about the drone’s intentions by clearly conveying the actions. A possible explanation, based on the qualitative responses, is that the arrows on the drone made it intuitive for participants to interpret each interaction stage and the drone’s motion. This aligns with prior work^25,31^ that highlighted the importance of arrow projections for ground robots and the use of arrow projections on head-mounted displays to communicate drone’s lateral movements. Our study makes a novel contribution by extending the arrow projection concept to displays mounted on drones, which was previously used to convey “emotions”^30,43^. We recommend drone designers use displays for motion cues and projection interfaces for drop-off spots and safety zones, reducing uncertainty and enhancing trust. This approach could benefit applications beyond delivery, such as law enforcement, cleaning, and emergency response, where drones can communicate motion and guide humans to safety. Future research should validate these designs across diverse HDI applications in public environments.

Among the three interfaces, lights were associated with the highest uncertainty and the lowest trust scores. Despite being perceived as simple and effective, participants reported difficulty with interpreting the light animations. These findings contrast with previous studies that studied indicator lights for robots^53^ and drones^24^ moving in lateral planes. An explanation, based on the qualitative feedback, is that the vertical positioning of the lights made it difficult for participants to distinguish between the top and bottom lights when the drone was positioned above eye level. This limitation could have hindered participants’ ability to associate the lights with familiar motor-vehicle signal models. Before recommending lights, future research should explore optimal light positioning to improve clarity and legibility in conveying motion cues of a robot operating in a vertical plane.

The display and the lights were attached to the drone’s face. Participants explained that the display and lights on the drone required them to look upward, particularly during the cable drop method when the drone hovered above eye level. Participants reported the potential visibility issues (e.g., glare) in bright sunlight and raises uncertainty and safety concerns. This raises an important limitation that was not considered in the previous HRI studies^24,30^, which did not account for the challenges of looking up to interpret signals.

Most participants felt certain about approaching the drop-off spot before the drone exited the scene when interfaces were present. Many indicated an intent to approach before the drone took off, and in some cases, even before the package was dropped, especially with the display or projection. These interfaces improve predictability of drone actions, reduce hesitation, and increase trust, which enables recipients to approach the drop-off spot earlier. This behavior may pose safety risks if recipients obstruct the drop-off spot and interfere with the drone’s operation. Future research should validate our findings in real-world settings and examine how close and at what moment recipients approach the drop-off spot during the interaction.

Our study focused on three visual interfaces with variations in symbology and animations for controllability. Based on the qualitative responses, participants recommended changing projection symbols, color-coding, and combining multiple interfaces (e.g., display and projection) to enhance clarity and attract users’ attention. As we found, some recipients might misinterpret the “H” symbol as indicating a drone landing rather than dropping a package with a suspended cable. We recommend replacing the symbol with explicit text or alternative symbols (e.g., “X”) to improve clarity about whether the drone is landing or the package is being dropped. Additionally, colors may carry intuitive meanings from traffic contexts^51,55^, potentially confusing users about egocentric (intent) versus allocentric (suggestion) cues, raising uncertainty and trust concerns. Future research should examine how colors influence the interpretation of drone intentions in public interactions.

Very small differences were observed between the delivery methods. The cable drop method was perceived as more understandable and predictable, particularly when no interfaces were present and recipients had to rely solely on the delivery method to interpret the drone’s intentions. Participants expressed safety concerns about propellers in proximity, which led to hesitation when approaching the drop-off spot during the landing method. Aligning with Bevins & Duncan^17,18^, humans tend to move away from drones undergoing altitude changes. On similar lines, Szafir et al.^23^ found that drones landing on the ground are perceived as less safe and less intuitive to interact with than drones following an arc trajectory without descending. Consistent with Lingam et al.^4^, participants felt safer with the cable drop method, as the drone hovered at 7 m, reducing propeller-related risks. However, concerns included reliability in bad weather, cable malfunctions, and interference from children or animals. Future research is recommended to further investigate the delivery methods by introducing variability in the delivery behavior and validate our findings.

## Considerations and limitations

We focused on HDI in a simulated public park under daylight and calm weather conditions. The experiment is controlled to investigate the effects of visual cues and minimize the influence of confounding variables such as environmental factors. While the current study setup improved experimental control, replicability of findings, and reduced both physical and perceived risks^42^, it limited ecological validity. The study is limited in replicating real-world conditions, including variations in ambient light and wind, the presence of glare, and the presence of external agents (e.g., kids or wildlife). These factors may increase users’ perceived uncertainties^9^ and may change perception of visual cues. For example, participants in our study reported that glare could reduce the visibility of the display from certain angles and that bright sunlight could diminish the legibility of information projected onto the ground. Such challenges with the real-world application could increase the feeling of uncertainty and raise the need for real-world evaluations. The effect sizes reported for the visual cues might differ in real-world settings for the reasons mentioned above. Future research should conduct naturalistic studies involving user interactions with drones to investigate how environmental factors and the presence of external agents influence recipients’ uncertainty and their perception of visual cues. Such studies would help assess how the proposed visual designs perform under complex, real-world public environments.

Considerable effort was made to calibrate the drone’s propeller noise and PID controllers to replicate the movements and sounds of a real drone. External factors, such as environmental noise or variations in the drone’s acceleration during ascent or descent in public spaces, may influence recipients’ perceptions. For example, a rapidly descending drone with loud propeller noise increases feelings of uncertainty and safety concerns^4^. This could influence the recipients to rely more on the drone’s movements and noise rather than on visual cues to interpret the drone’s intentions. Future research should examine how the visual cues perform under real-world conditions, including environmental noise and variable drone acceleration during ascent and descent in public spaces.

Furthermore, the videos were presented from the perspective of a recipient positioned at a fixed distance from the drone to ensure experimental control and scenario repeatability. In real-world situations, however, recipients may move and position themselves at varying distances and viewing angles. Such proxemic behavior could influence their sense of uncertainty. For instance, a recipient may perceive a descending drone as unsafe and move farther away, which could reduce the visibility of the visual interfaces, particularly the lights. Conversely, a recipient might move closer or change their position to obtain a better viewing angle, potentially decreasing uncertainty about drone intentions. Future research should examine how recipients naturally position themselves around drones in the presence of visual cues and how these behavior affect their sense of uncertainty.

## Conclusions

Our study highlights the importance of conveying explicit information about the intentions of a robot (i.e., drone) flying and delivering a package in a simulated public space, enabling recipients to predict the robot’s actions and approach the drop-off spot with certainty and trust. While the positioning of the light interface posed challenges for clarity, both display and projection interfaces reduced uncertainty and improved trust by conveying information on drone actions and drop-off spots. Projection was particularly recommended, as it was favored for marking the drop-off spot on the ground and a safety boundary. As the study provides observations in a simulated environment, we suggest that the cues be adapted through further research and validated in real-world settings before being applied in public spaces. We hope that the cues are beneficial not only to delivery drones but also to drones operating in public areas for tasks such as law enforcement, cleaning, and emergency response. Despite the need for further real-world validation, this study contributes to the design of robots operating in vertical space by investigating how the designs and placement of visual cues affect recipient perceptions of uncertainty and trust in a public-space context.

## Supplementary Information

Below is the link to the electronic supplementary material.

Supplementary Material 1

## Data Availability

The datasets analyzed in the current study are available in the supplementary material.
