# Supplementary material for: Delivery from the sky: investigating visual cues to communicate robot intentions in simulated public spaces
Source: Sci Rep. 2026 Mar 4;16:12094. doi: 10.1038/s41598-026-36451-z (PMC13076782; doi:10.1038/s41598-026-36451-z)
Supplement: Supplementary file 1 — Supplementary Material 1 [file 41598_2026_36451_MOESM1_ESM.zip › Supplementary_results.pdf]

# Supplementary results

Figure S1 presents participant-level uncertainty scores (N = 150) across interface conditions, averaged over delivery methods. The Baseline condition exhibited the highest uncertainty ratings with greater variability compared to the three interfaces. While none of the interfaces reached the highest uncertainty levels of the Baseline, Lights received higher uncertainty scores and greater variation than Display and Projection. However, the difference between Display and Projection was not visibly substantial.

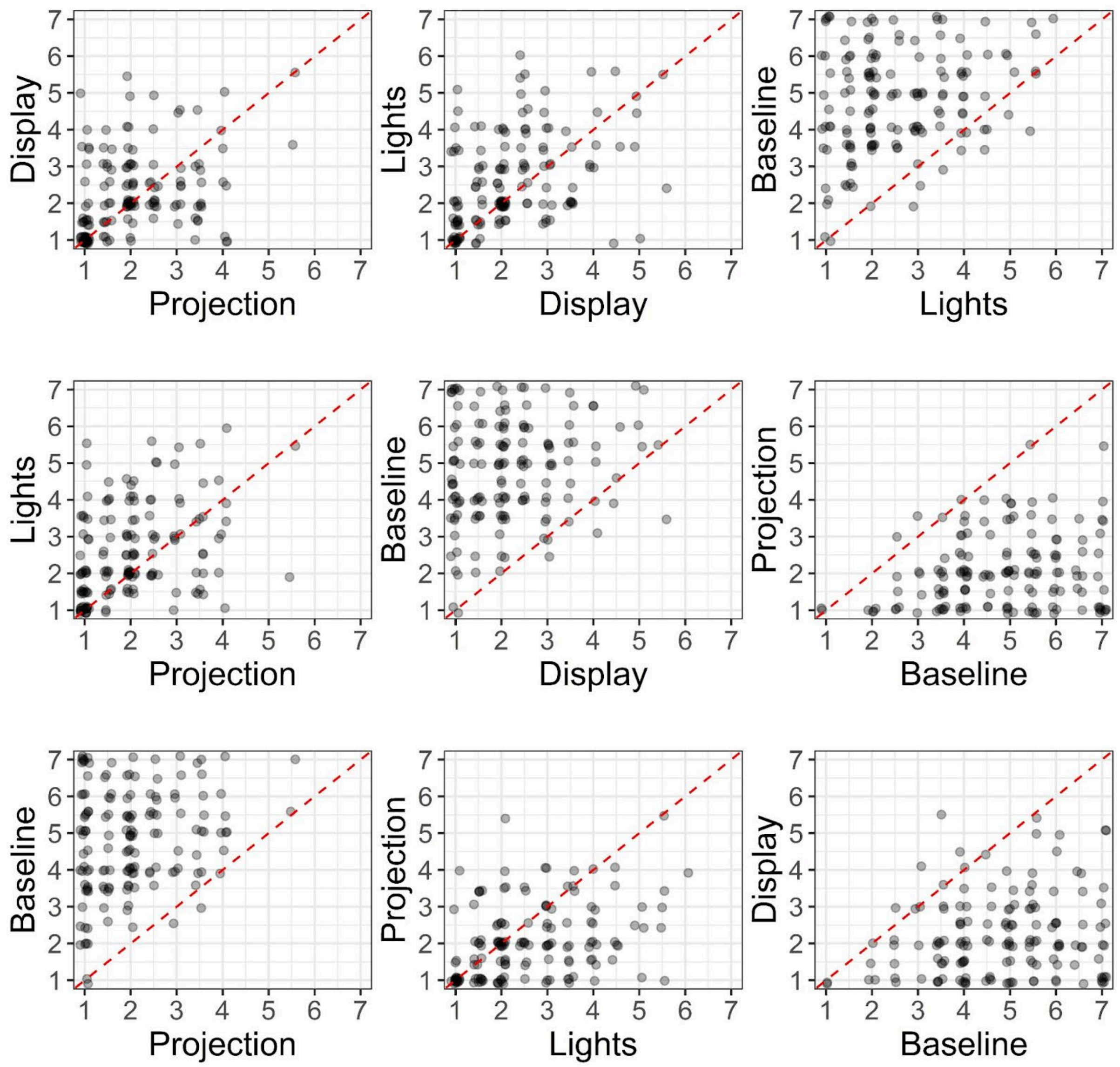


Figure S1: Uncertainty scores for the sample (N = 150), represented by black dots, comparing the four interface conditions (Baseline, Display, Lights, and Projection), averaged over delivery methods. The red dotted line represents a 45-degree reference line, indicating equal ratings between the compared interfaces.
